# An Innovative High-Precision Scheme for a GPS/MEMS-SINS Ultra-Tight Integrated System

**DOI:** 10.3390/s19102291

**Published:** 2019-05-17

**Authors:** Qunsheng Li, Yan Zhao

**Affiliations:** 1 School of Instrument Science and Opto-electronics Engineering, Beihang University, Beijing 100191, China; zhaoyan@buaa.edu.cn; 2 Luoyang Optoelectro Technology Development Center, Luoyang 471000, China

**Keywords:** MEMS-SINS, global positioning system, ultra-tight integration, carrier phase differential, tracking loop

## Abstract

The Doppler-assisted error provided by a low-precision microelectromechanical system (MEMS) strapdown inertial navigation system (SINS) increases rapidly. Therefore, the bandwidth of the tracking loop for a global positioning system (GPS)/MEMS-SINS ultra-tight integration system is too narrow to track Doppler shift. GPS measurement error is correlated with the MEMS-SINS velocity error when the Doppler-assisted error exists, leading to tracking loop lock loss. The estimated precision of the integrated Kalman filter (IKF) also decreases. Even the integrated system becomes unstable. To solve this problem, an innovative GPS/MEMS-SINS ultra-tight integration scheme based on using high-precision carrier phase measurements as the IKF measurements is proposed in this study. By assisting the tracking loop with time-differenced carrier phase (TDCP) velocity, the carrier loop noise bandwidth and code correlator spacing are reduced. The tracking accuracies of the carrier and code are increased. The navigation accuracy of GPS/MEMS-SINS ultra-tight integration is further improved.

## 1. Introduction

The error characteristics of global positioning systems (GPSs) and strapdown inertial navigation systems (SINSs) are highly complementary. GPS/SINS integrated systems have the advantages of both GPS and SINS [1]. According to different data fusion strategies, the GPS/SINS integrated system can be implemented in three modes: with loose, tight, and ultra-tight integration [2]. Ultra-tight integration has become the main direction of GPS/SINS integrated systems due to its superior dynamic performance and anti-jamming capability [3]. GPS/MEMS-SINS integrated systems are important in military and commercial applications due to their low cost, small volume, low power dissipation, lightweightness, and high reliability [4]. In GPS/SINS ultra-tight integration, both the tracking loop filter output and the Doppler-assisted information are used to generate control instructions for the numerically controlled oscillator (NCO) [5]. Consequently, the dynamic performance of the GPS receiver is improved by reducing dynamic stress, and its loop thermal noise is restrained by reducing the loop bandwidth [6]. However, the response of the loop filter to tracking error is limited. The tracking loop is more dependent on the Doppler-assisted information for controlling the NCO, and the loop is more sensitive to the Doppler-assisted error [7,8]. There is a relationship between the accuracy of Doppler-assisted information and the MEMS-SINS device quality. As a consequence, low-precision MEMS-SINS-assisted information would mislead the tracking loop and result in tracking loop lock loss. This causes GPS measurement error to be correlated with the MEMS-SINS velocity error, and the correlation results in the decrease in the estimate precision of the integrated Kalman filter (IKF).

The GPS receiver uses a pseudo-range for positioning in ordinary conditions; the horizontal position error is about 10 m, the vertical position error is about 15 m, and the velocity error is about 0.2 m/s [9]. With the requirement for increased navigation accuracy, carrier phase measurements h been generally accepted in the microelectromechanical system [10]. Lee presented an effective carrier-smoothed-code filter for kinematic differential positioning [11]. The vehicle experiment result demonstrated that the position error was below 0.5 m. Ding proposed an improved time-differenced carrier phase (TDCP) velocity estimation approach [12]. The velocity error of this proposed algorithm using kinematic field test data is about 2.9 mm/s. Wendel proposed an approach to enhance the performance of tightly coupled GPS/INS systems using the TDCP in the IKF [13]. The hardware-in-the-loop test showed that the missile velocity error using this method is about 0.04 m/s and the attitude error is about 0.002 rad. Moafipoor presented a filter update method based on GPS carrier phase velocity calibration in the GPS/INS integration system to ensure the availability and continuity of the navigation solution [14]. The proposed method has a position error of about 14 cm and velocity error of about 3 mm/s.

Thus, using high-precision GPS TDCP velocity estimation to aid the tracking loop would reduce the carrier Doppler frequency error and the correlation between the GPS measurement error and the MEMS-SINS velocity error. Integrating high-precision GPS carrier phase measurements and MEMS-SINS would improve the accuracy of the GPS/MEMS-SINS integration. Therefore, this paper presents an innovative scheme for a carrier phase differential GPS/MEMS-SINS integrated system. TDCP velocity-assisted information is used to overcome the problem of large Doppler-assisted error caused by low-precision MEMS-SINS. High-precision TDCP velocity and carrier-smoothed pseudo-range (CSP) are used as the IKF measurements to enhance the dynamic performance, anti-jamming capability, and accuracy of navigation.

## 2. Scheme of the Proposed GPS/MEMS-SINS Ultra-Tight Integration

**Figure 1:**
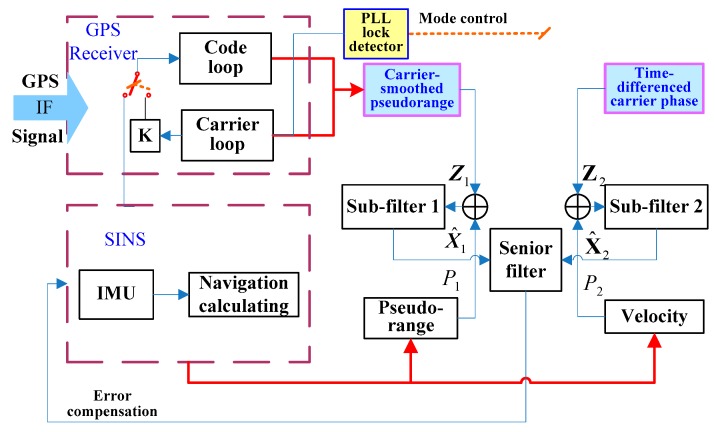
Innovative scheme for the GPS/MEMS-SINS ultra-tight integrated system. GPS: global positioning system; MEMS-SINS: microelectromechanical system–strapdown inertial navigation system; IF: intermediate frequency; K:switch; PLL: phase lock loop; IMU: inertial measurement unit; Z:measurement; P:variance matrix.

The Kalman filter is the data fusion method generally used in GPS/SINS integrated systems. The estimate precision of the IKF state vector is related to the accuracy of the model and measurements [15]. To improve the accuracy of GPS/MEMS-SINS ultra-tight integration, by assisting the tracking loop with TDCP velocity, an innovative GPS/MEMS-SINS ultra-tight integration scheme based on high-precision carrier phase measurement as the IKF measurement is proposed. The system configuration of the proposed integration in outlined in Figure 1.

The proposed scheme adopts TDCP velocity and CSP as the IKF measurements to estimate the state vector and to correct SINS errors. Phase lock loop (PLL) lock detector output is used as the operating mode control parameter to control GPS-assisted information. In normal working conditions, the TDCP velocity aids the PLL. The delay lock loop (DLL) is aided by low-noise-level PLL frequency estimation transformed by a scale factor. In complex working conditions, if carrier phase cycle slips occur, the TDCP velocity would be inaccurate. The innovative ultra-tight integration switches to MEMS-SINS-assisted PLL, and the PLL-assisted DLL mode is switched to MEMS-SINS-assisted DLL. The robustness of the innovative ultra-tight integration is enhanced during severe working conditions.

PLL is sensitive to the Doppler-assistederror, and it is the weak link in GPS/SINS ultra-tight integration. TDCP velocity is used to aid the GPS tracking loop to reduce the Doppler-assistederror, so the control instructions for the NCO could be more accurate. The PLL tracking accuracy is improved under highly dynamic and severe jamming conditions. The probability of loss lock decreases. Adopting TDCP velocity and CSP as the IKF measurements could improve the accuracy of IKF estimation. There are different updating rates and noise characteristics between the measurements. To avoid the high-precision TDCP velocity being obscured by the high noise level of the pseudo-range, two parts of the measurements are separated by considering the different updating rates. Considering the configuration of the federated filter, the innovative GPS/MEMS-SINS ultra-tight integration isolates the measurement noise of the CSP and TDCP velocity to take advantage of the high-precision measurement.

In this study, based on the above literature, TDCP was used to improve the speed measurement accuracy, CSP was used to improve the pseudo-distance measurement accuracy, a system solution was created on the basis of low-cost and low-precision MEMS/SINS, and a laboratory semi-physical simulation experiment and land vehicle experiment were conducted to further verify the feasibility of the scheme.

## 3. Establishment of the High-Precision GPS Measurement Model

### 3.1. Carrier-Smoothed Pseudo-Range Measurement Model

**Figure 2:**
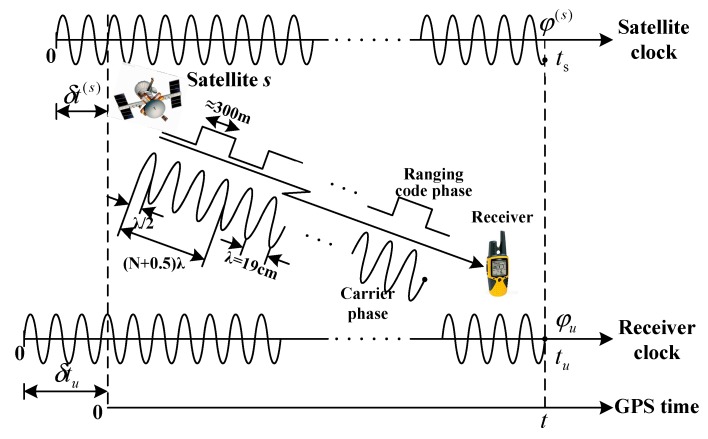
Schematic of the principle of the carrier-smoothed pseudo-range.

In the past, the pseudo-range was regarded as the most important basic measurement of a GPS receiver. With the increasing need for better navigation accuracy, carrier phase measurement has received more attention. The measurement noise in carrier phase measurements is much lower than the measurement noise in the pseudo-range. Because carrier phase measurements are smoothed and highly precise, the multipath error in carrier phase measurements is much lower than the multipath error in the pseudo-range. However, integer ambiguity is the key problem limiting the application of carrier phase measurements. The convergence time for the ambiguity is generally about 30 min [16]. However, with continuous carrier tracking, the integer ambiguity remains constant. Therefore, the integer ambiguity is eliminated by the difference between successive epochs of the carrier phase. These high-precision carrier phase measurements could be used to smooth the pseudo-range without integer ambiguity. The principle of carrier smoothed pseudo-range is shown in Figure 2.

$\varphi^{\left(s\right)}$ is the phase of L1 carrier launched by the satellite, and $\varphi_{u}$b is the phase of L1 carrier received by the receiver, $t_{s}$ is the time of the satellite, $t_{u}$ is the time of the receiver and $t_{}$ is GPS time.

In GPS L1 signal carrier frequency, the pseudo-range and carrier models are presented as follows at epoch time *k*:

(1) $$\rho_{k}=r_{k}+c\left(\delta t_{u,k}-\delta t_{k}^{\left(s\right)}\right)+I_{k}+T_{k}+O_{k}+M_{\rho ,k}+\varepsilon_{\rho ,k}$$

(2) $$\phi_{k}=\lambda^{-1}{\left[r_{k}+c\left(\delta t_{u,k}-\delta t_{k}^{\left(s\right)}\right)-I_{k}+T_{k}+O_{k}+M_{\phi ,k}\right]}^{}+N+\varepsilon_{\phi ,k}$$

where $\rho_{k}$ is the pseudo-range measurement; $\phi_{k}$ is the carrier phase measurement; $r_{k}$ is the distance between the satellite and the GPS receiver; λ is the wavelength of the L1 carrier frequency; $I_{k}$ and $T_{k}$ are the ionosphere and troposphere delay equivalent range errors that belong to the transmission error, respectively; $\delta t_{u}$ and $\delta t^{\left(s\right)}$ are the GPS receiver clock error and satellite clock error, respectively; $O_{k}$ is satellite orbit error that belongs to the control segment error; M and ε are the multipath error and measurement noise, respectively; N is the integer ambiguity; and *c* is the speed of light in a vacuum.

As the carrier has been locked without cycle slips, the integer ambiguity of the carrier phase measurement remains constant at any time. The pseudo-range and carrier are respectively subtracted from successive epochs as expressed below:

(3) $$\begin{array}{ll} \rho_{k}-\rho_{k-1} & =\Delta \rho_{k}=r_{k}-r_{k-1}+c{\left[\left(\delta t_{u,k}-\delta t_{u,k-1}\right)-\left(\delta t_{k}^{\left(s\right)}-\delta t_{k-1}^{\left(s\right)}\right)\right]}^{}+I_{k}-I_{k-1}\\ & +T_{k}-T_{k-1}+O_{k}-O_{k-1}+M_{\rho ,k}-M_{\rho ,k-1}+\varepsilon_{\rho ,k}-\varepsilon_{\rho ,k-1}\\ & =\Delta r_{k}+c\left(\Delta \delta t_{u,k}-\Delta \delta t_{k}^{\left(s\right)}\right)+\Delta I_{k}+\Delta T_{k}+\Delta O_{k}+\Delta M_{\rho ,k}+\Delta \varepsilon_{\rho ,k} \end{array}$$

(4) $$\begin{array}{ll} \lambda \cdot \left(\phi_{k}-\phi_{k-1}\right) & =\lambda \cdot \Delta \phi_{k}=r_{k}-r_{k-1}+c{\left[\left(\delta t_{u,k}-\delta t_{u,k-1}\right)-\left(\delta t_{k}^{\left(s\right)}-\delta t_{k-1}^{\left(s\right)}\right)\right]}^{}-\left(I_{k}-I_{k-1}\right)\\ & +T_{k}-T_{k-1}+O_{k}-O_{k-1}+M_{\phi ,k}-M_{\phi ,k-1}+\lambda \cdot \left(\varepsilon_{\phi ,k}-\varepsilon_{\phi ,k-1}\right)\\ & =\Delta r_{k}+c\left(\Delta \delta t_{u,k}-\Delta \delta t_{k}^{\left(s\right)}\right)-\Delta I_{k}+\Delta T_{k}+\Delta O_{k}+\Delta M_{\phi ,k}+\lambda \cdot \Delta \varepsilon_{\phi ,k} \end{array}$$

where the integer ambiguity of *N* would be counteracted, $\Delta \phi_{k}$ is the integrated Doppler from epoch *k* − 1 to *k*; $\Delta \rho_{k}$ is the pseudo-range measurement change from epoch *k* − 1 to *k*; $\Delta r_{k}$ is the distance change between the satellite and the GPS receiver from epoch *k* − 1 to *k*; $\Delta I_{k}$ and $\Delta T_{k}$ are ionosphere and troposphere delay equivalent range error change from epoch *k* − 1 to *k*, respectively; $\Delta \delta t_{u}$ and $\Delta \delta t^{\left(s\right)}$ are the GPS receiver clock error and satellite clock error change from epoch *k* − 1 to *k*, respectively; $\Delta O_{k}$ is the satellite orbit error change from epoch *k* − 1 to *k*; ΔM and Δε are the multipath error change and measurement noise change from epoch *k* − 1 to *k*, respectively; and λ is the wavelength of the L1 carrier frequency. Thus, the pseudo-range and carrier phase variation can be combined into a kind of smoothed measurement without integer ambiguity. Using the modified Hatch filter [17], the recurrence formula is as follows:

(5) $$\rho_{s,k}=\frac{1}{m}\rho_{k}+\frac{m-1}{m}\left[\rho_{s,k-1}+\lambda \left(\phi_{k}-\phi_{k-1}\right)\right]$$

Equation (5) is the smoother equation of the carrier phase-smoothed pseudo-range measurement. The smoother output $\rho_{s,k}$ is called the carrier-smoothed pseudo-range (CSP) at epoch *k*, *m* is the smoothing time constant, and *m* is generally in the range of 20 to 100.

If carrier phase lock loss or cycle slips occur, then the smoother must be reset. The first pseudo-range measurement of the GPS receiver when the carrier phase locked is used to initialize $\rho_{s,k}$ is expressed as:

(6) $$\rho_{s,1}=\rho_{1}$$

### 3.2. Time-Differenced Carrier Phase Measurement Model

**Figure 3:**
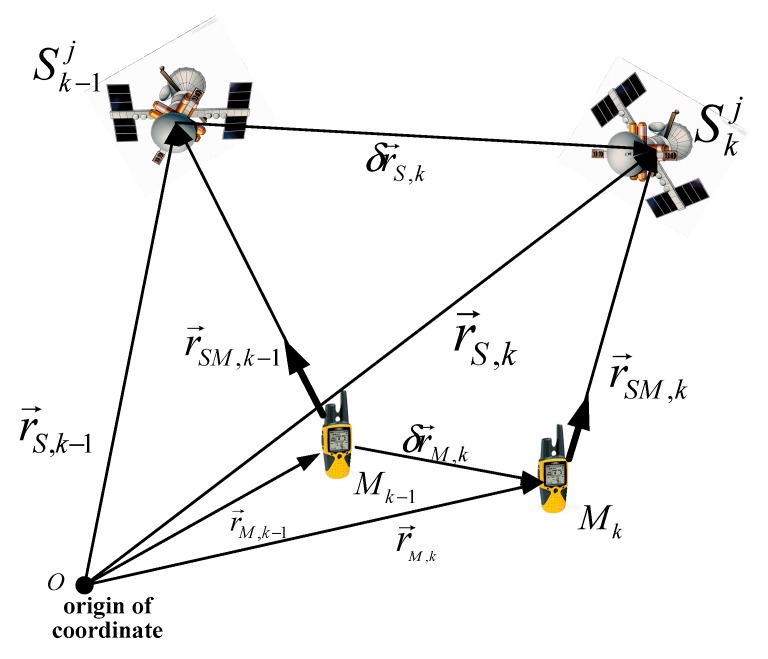
Schematic of the principle of the time-differenced carrier phase (TDCP) process. $S_{k-1}^{j}$ and $S_{k}^{j}$ are satellite numbered *j* at epoch *k* − 1 and *k*, ${\vec{r}}_{S,k-1}$ and ${\vec{r}}_{S,k}$ are position of satellite numbered *j* at epoch *k* − 1 and *k* − 1, $\delta {\vec{r}}_{S,k}$ is the satellite flight distance from epoch *k* − 1 to *k*, $M_{k-1}$m and $M_{k}$n are GPS receiver at epoch *k* − 1 and *k*, ${\vec{r}}_{M,k-1}$ and ${\vec{r}}_{M,k}$ are position of GPS receiver at epoch *k* − 1 and *k*, $\delta {\vec{r}}_{M,k}$ is the GPS receiver distance moved from epoch *k* − 1 to *k*, ${\vec{r}}_{SM,k-1}$ and ${\vec{r}}_{SM,k}$ are the distances between the GPS receiver and the satellite at epoch *k* − 1 and *k*.

Carrier phase measurement is a method of determining an accurate signal propagation distance by measuring the carrier along the carrier propagation path [18]. The integer ambiguity is a constant with continuous carrier tracking. Consequently, the single-difference measurement without integer ambiguity can be determined by the difference between successive epochs of the carrier phase, and then the velocity measurement can be determined. The TDCP working principle is shown in Figure 3.

According to Equation (2), the equation for TDCP measurement can be derived. Considering that multipath error and measurement noise are both types of random noise, they can be merged into one term, ξ. The rewritten equation of the carrier phase measurement is:

(7) $$\lambda \cdot \phi_{k}=r_{k}+c\left(\delta t_{u,k}-\delta t_{k}^{\left(s\right)}\right)-I_{k}+T_{k}+O_{k}+\lambda \cdot N+\xi$$

The carrier phase was subtracted during successive epochs as follows:

(8) $$\lambda \cdot \Delta \phi_{k}=\Delta r_{k}+c\left(\Delta \delta t_{u,k}-\Delta \delta t_{k}^{\left(s\right)}\right)-\Delta I_{k}+\Delta T_{k}+\Delta O_{k}+\Delta \xi$$

Since the error variable quantities of the ionosphere, troposphere, satellite clock, and satellite orbit are less than 2.5 mm, these errors are in the same level as the carrier phase measurement error of the GPS receiver. Therefore, these four kinds of errors are eliminated by the difference between successive epochs. Δξ is the multipath error and measurement noise change from epoch *k* − 1 to *k*.

$\tilde{\phi }$ is defined as the carrier phase measurement without ionosphere, troposphere, satellite clock, and satellite orbit errors [19]:

(9) $$\lambda \cdot \Delta {\tilde{\phi }}_{k}=\lambda \cdot \Delta \phi_{k}+\Delta I_{k}-\Delta T_{k}-\Delta O_{k}+c\cdot \Delta \delta t_{k}^{\left(s\right)}\cdot =\Delta r_{k}+c\cdot \delta t_{u,k}+\Delta \xi$$

where $\Delta {\tilde{\phi }}_{k}$ is the carrier phase measurement change from epoch *k* − 1 to *k*. According to the position relationship between the satellite and the GPS receiver shown in Figure 3 during two successive GPS measurements, Δr can be expressed as:

(10) $$\begin{array}{ll} \Delta r_{k} & ={\vec{r}}_{SM,k}-{\vec{r}}_{SM,k-1}=\left({\vec{r}}_{S,k}-{\vec{r}}_{M,k}\right)\cdot e_{k}^{\left(s\right)}-\left({\vec{r}}_{S,k-1}-{\vec{r}}_{M,k-1}\right)\cdot e_{k-1}^{\left(s\right)}\\ & ={\vec{r}}_{S,k}\cdot e_{k}^{\left(s\right)}-{\vec{r}}_{S,k-1}\cdot e_{k-1}^{\left(s\right)}-{\vec{r}}_{M,k}\cdot e_{k}^{\left(s\right)}+{\vec{r}}_{M,k-1}\cdot e_{k-1}^{\left(s\right)} \end{array}$$

where $e_{k}^{\left(s\right)}$ represents the line-of-sight (LOS) unit vector from the GPS receiver to the observed satellite at epoch *k*. 

As shown in Figure 3, the GPS receiver position ${\vec{r}}_{M,k}$ can be described as:

(11) $${\vec{r}}_{M,k}={\vec{r}}_{M,k-1}+\delta {\vec{r}}_{M,k}$$

Equation (10) can be expressed as:

(12) $$\begin{array}{ll} \Delta r_{k} & ={\vec{r}}_{S,k}\cdot e_{k}^{\left(s\right)}-{\vec{r}}_{S,k-1}\cdot e_{k-1}^{\left(s\right)}-{\vec{r}}_{M,k}\cdot e_{k}^{\left(s\right)}+{\vec{r}}_{M,k-1}\cdot e_{k-1}^{\left(s\right)}\\ & =\left({\vec{r}}_{S,k}\cdot e_{k}^{\left(s\right)}-{\vec{r}}_{S,k-1}\cdot e_{k-1}^{\left(s\right)}\right)-\left({\vec{r}}_{M,k-1}\cdot e_{k}^{\left(s\right)}-{\vec{r}}_{M,k-1}\cdot e_{k-1}^{\left(s\right)}\right)-\delta r_{M,k}\cdot e_{k}^{\left(s\right)} \end{array}$$

After simplified treatment, Equation (12) can be rewritten as:

(13) $$\Delta r_{k}=S_{Dop}^{\left(s\right)}-Geo_{k,k-1}-\delta r_{M,k}\cdot e_{k}^{\left(s\right)}$$

Figure 3 shows that $S_{Dop}^{\left(s\right)}$ is the distance variation from epoch *k* − 1 to epoch *k*. $Geo_{k,k-1}$ is the variation in the geometric relation of the observed satellite and the GPS receiver caused by the change of the LOS vector. 

Equation (13) is substituted into Equation (9) as follows:

(14) $$\lambda \cdot \Delta {\tilde{\phi }}_{k}^{\left(s\right)}=S_{Dop}^{\left(s\right)}-Geo_{k,k-1}-\delta r_{M,k}\cdot e_{k}^{\left(s\right)}+c\cdot \delta t_{u,k}+\Delta \xi$$

where $S_{Dop}^{\left(s\right)}$ is calculated by the observed satellite and the LOS vector. The satellite position is calculated by the navigation message. $Geo_{k,k-1}$ is calculated by the GPS receiver position, the satellite position, and the LOS vector. $\tilde{\tilde{\phi }}$ is the carrier phase measurement in which $\tilde{\phi }$ eliminates the influence of $S_{Dop}^{\left(s\right)}$ and $Geo_{k,k-1}$ as follows:

(15) $$\lambda \cdot \Delta {\tilde{\tilde{\phi }}}_{k}^{\left(s\right)}=\lambda \cdot \Delta {\tilde{\phi }}_{k}-S_{Dop}^{\left(s\right)}+Geo_{k,k-1}$$

Equation (15) is substituted into Equation (14) to obtain the measurement as shown:

(16) $$\lambda \cdot \Delta {\tilde{\tilde{\phi }}}_{k}^{\left(s\right)}=-\delta r_{M,k}\cdot e_{k}^{\left(s\right)}+c\cdot \delta t_{u,k}+\Delta \xi$$

Therefore, the measurements of different satellites can be written in matrix form as

(17) $$Z=H\cdot \left[\begin{array}{c} \delta r_{M,k}\\ \delta t_{u,k} \end{array}\right]$$

where $\delta r_{M,k}$ is the distance variation from epoch *k* − 1 to epoch *k*, $\delta t_{u,k}$ is the GPS receiver clock error, and the measurement matrix ***H*** and the measurement ****Z**** are

$$H=\left[\begin{array}{cc} e_{k}^{\left(1\right)}{}^{T} & 1\\ e_{k}^{\left(2\right)}{}^{T} & 1\\ \vdots & 1\\ e_{k}^{\left(N\right)}{}^{T} & 1 \end{array}\right]\;\mathrm{and}\;Z=\left[\begin{array}{c} \lambda \cdot \Delta {\tilde{\tilde{\phi }}}_{k}^{\left(1\right)}\\ \lambda \cdot \Delta {\tilde{\tilde{\phi }}}_{k}^{\left(2\right)}\\ \vdots \\ \lambda \cdot \Delta {\tilde{\tilde{\phi }}}_{k}^{\left(N\right)} \end{array}\right],\;N\geq 4$$

Therefore, the velocity of the GPS receiver can be estimated by the minimum mean square error:

(18) $$\left[\begin{array}{c} \delta r_{M,k}\\ \delta t_{u,k} \end{array}\right]={\left(H^{T}\cdot H\right)}^{-1}H^{T}\cdot Z$$

## 4. Innovative SINS/GPS Ultra-Tight Integration Model

### 4.1. Modeling and Analysis of TDCP Velocity-Assisted PLL Tracking

**Figure 4:**
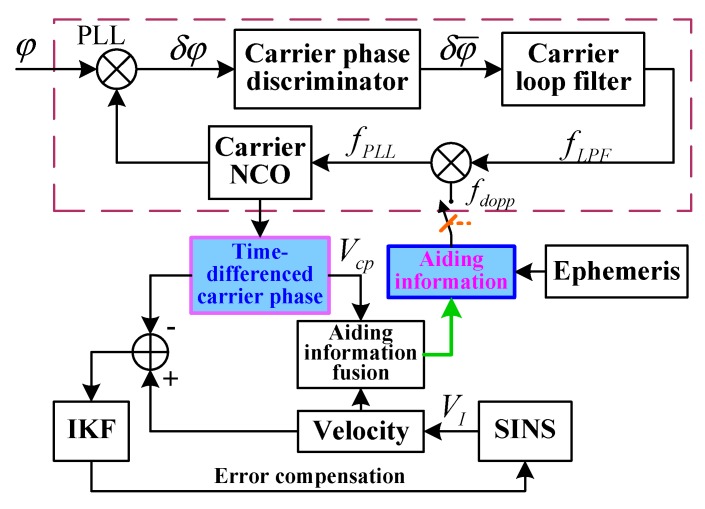
Configuration of TDCP velocity-assisted PLL tracking. NCO: numerically controlled oscillator; $f_{dopp}$ is the Doppler frequency, $f_{LPF}$ is output of loop filter, $f_{PLL}$ is Control frequency of NCO, $V_{cp}$ is TDCP velocity, $V_{I}$ is velocity of SINS.

The carrier loop is the weak link in a GPS receiver. The dynamic effect of the carrier loop easily causes carrier loop lose lock. The TDCP velocity-assisted PLL helps to eliminate most of the dynamic stress of the signal. The capability of tracking the dynamic signal is enhanced and the probability of carrier loop lock loss is reduced. In addition, the main error source of PLL includes thermal noise, which can be reduced by narrowing the noise bandwidth. However, narrowing the noise bandwidth will also increase the dynamic stress error. The assisted information can solve this problem well. The introduction of TDCP velocity-assisted can increase the loop equivalent bandwidth and reduce the dynamic stress error. To ensure the dynamic tracking range of PLL, the loop filter bandwidth can be reduced to restrain the thermal noise. The configuration of TDCP velocity-assisted PLL tracking is shown in Figure 4.

Supposing that the position and velocity of the GPS satellite $S^{j}$ in the Earth-centered Earth-fixed (ECEF) frame are $X_{S}^{j}$ and $V_{S}^{j}$, respectively, and the position and velocity of GPS receiver in the ECEF frame are $X_{r}$ and $V_{cp}$, respectively, then the Doppler-assisted frequency is

(19) $$f_{dopp}=\frac{f_{L1}}{c}\cdot V_{aid}=\frac{f_{L1}}{c}\cdot (V_{cp}-V_{S}^{j})\cdot \frac{\left(X_{r}-X_{S}^{j}\right)}{\left\|X_{r}-X_{S}^{j}\right\|}=\frac{f_{L1}}{c}(V_{cp}-V_{S}^{j})\cdot {\vec{L}}_{i}$$

where ${\vec{L}}_{i}$ is the direction cosine matrix (DCM) between satellites and the GPS receiver from the Earth-centered inertial (ECI) frame to the ECEF frame, *c* is the speed of light in a vacuum, $f_{L1}$ is the GPS signal L1 carrier frequency, and $V_{aid}$ is the assisted velocity.

Transforming the assisted velocity error to the Doppler frequency error is achieved as follows:

(20) $$\delta f_{dopp}=\frac{f_{L1}}{c}\cdot \delta V_{aid}=\frac{f_{L1}}{c}\cdot \delta V\cdot {\vec{L}}_{i}$$

where $\delta f_{dopp}$ is the Doppler frequency error, $\delta V_{aid}$ is the assisted velocity error, and δV is the velocity estimation error of a SINS.

**Figure 5:**
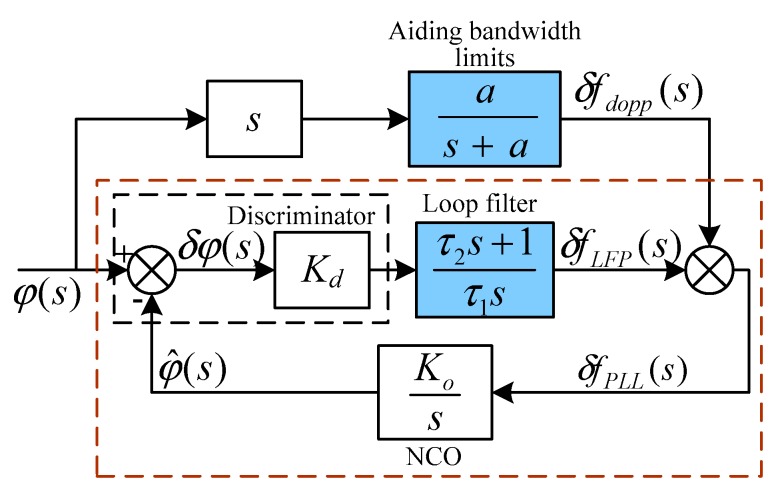
Model of TDCP velocity-assisted PLL tracking. Where φ is the phase of the input carrier signal, $\hat{\varphi }$ is the local carrier signal phase, δφ is the phase error between the local carrier and the input carrier, $\delta f_{dopp}$ is the Doppler-assisted error, $\delta f_{LFP}$ is the loop filter output, and $\delta f_{PLL}$ is the PLL frequency-tracking error. $G(s)=\frac{a}{s+a}$ is the low-pass filter used to limit the bandwidth of SINS. The loop filter is an ideal integral filter, $F(s)=\frac{\tau_{2}s+1}{\tau_{1}s}$, where $\tau_{1}$ and $\tau_{2}$ are the time constants of the filter, $K_{d}$ is the gain of the discriminator, and $K_{o}$ is the gain in NCO.

The error model of TDCP velocity-assisted PLL tracking is shown in Figure 5.

According to the loop structure, the closed-loop transfer function is

(21) $$H(s)=\frac{\hat{\varphi }(s)}{\varphi (s)}=\frac{\frac{K_{o}a}{s+a}+K_{PLL}\frac{\tau_{2}+1}{\tau_{1}}}{1+K_{PLL}\frac{\tau_{2}+1}{\tau_{1}}}$$

where $K_{PLL}=K_{o}K_{d}$ is the gain of the loop filter. Since the assisted information can extend the tracking loop bandwidth, the loop filter sets a low bandwidth to track the remaining frequency error, $\delta f_{dopp}$.

When the carrier loop is locked, the carrier phase error is expressed as

(22) $$\delta \varphi (s)=\varphi (s)-\hat{\varphi }(s)$$

After carrier phase error filtering, the filter output is

(23) $$\delta f_{LPF}(s)=K_{d}\frac{\tau_{2}s+1}{\tau_{1}s}\cdot \delta \varphi (s)$$

After transformation into the time-domain, Equation (23) is expressed as

(24) $$\delta {\dot{f}}_{LPF}=K_{d}\frac{\tau_{2}\delta \dot{\varphi }+\delta \varphi }{\tau_{1}}$$

The PLL frequency-tracking error $\delta f_{PLL}$ is the sum of the loop filter output $\delta f_{LPF}$ and the Doppler-assisted error $\delta f_{dopp}$:

(25) $$\delta f_{PLL}=\delta f_{LPF}+\delta f_{dopp}$$

The variation rate of the carrier phase tracking error $\delta \dot{\varphi }$ caused by $\delta f_{PLL}$ is

(26) $$\delta \dot{\varphi }=2\pi (\delta f_{LPF}+\frac{f_{L}}{c}\delta V^{T}\cdot {\vec{L}}_{i})$$

Equation (26) is substituted into Equation (24) to obtain the PLL error equation as follows:

(27) $$\delta {\dot{f}}_{LPF}=K_{d}\cdot \left[2\pi \frac{\tau_{2}}{\tau_{1}}\left(\delta f_{LPF}+\delta f_{dopp}\right)+\frac{\delta \varphi }{\tau_{1}}\right]$$

The TDCP velocity-assisted PLL mode includes an assisted loop and a carrier tracking loop. In the assisted loop, TDCP velocity is considered as assisting information to eliminate the dynamic stress in the carrier loop. In the carrier tracking loop, according to the output of the assisting frequency and loop filter, NCO modulates the carrier frequency. PLL only tracks the remaining frequency error, $\delta f_{dopp}$. The anti-jamming capability of PLL is enhanced by modulating the loop filter parameter to reduce the loop bandwidth.

### 4.2. Modeling and Analysis of PLL-Assisted DLL Tracking

**Figure 6:**
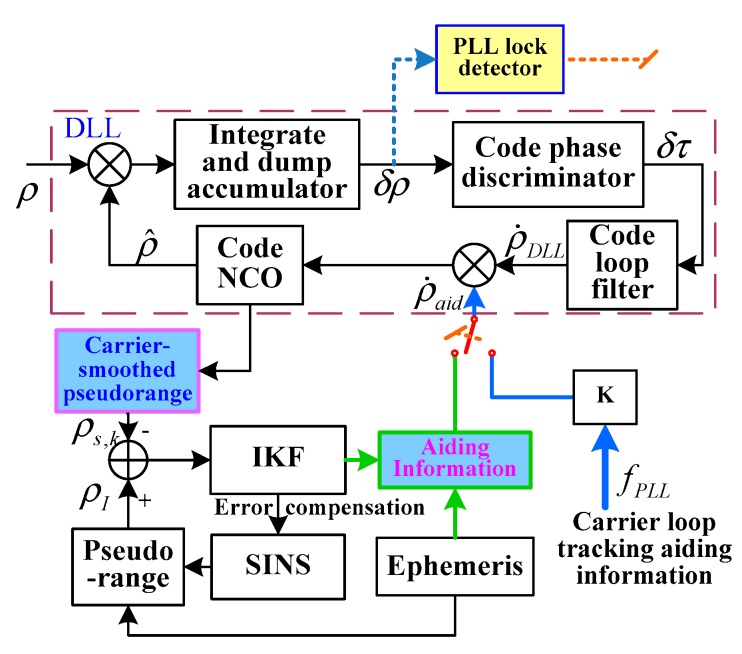
Model of TDCP velocity-assisted DLL tracking. Where $\rho_{I}$ is the pseudo-range calculated by SINS, $\rho_{s,k}$ is the carrier phase measurement smoothed pseudo-range, ρ is the pseudo-range measured by GPS, $\hat{\rho }$ is the pseudo-range estimated by the receiver loop, δρ is the pseudo-range error between the GPS measurement and receiver loop estimation, $f_{PLL}$ is the carrier loop tracking aiding frequency, δτ is the phase difference of the receiver pseudo-random code, ${\dot{\rho }}_{DLL}$ is the pseudo-range rate calculated by the receiver loop, and ${\dot{\rho }}_{aid}$ is the pseudo-range rate for aiding DLL.

To prevent the code loop from being tainted by the decrease in carrier tracking performance in complicated working conditions, two assisted modes were designed to help the code loop in the innovative ultra-tight integration. The two assisted modes are switched by judging whether the carrier loop is locked. If the carrier loop is locked, the code DLL is aided by PLL. Instead, the assisted mode is switched to MEMS-SINS-assisted DLL to eliminate the burden of DLL tracking the vehicle dynamics. The configuration of TDCP velocity-assisted DLL tracking is depicted in Figure 6.

The principle of assisted DLL involves using PLL frequency estimation to assist the code loop. The PLL loop filter output $f_{PLL}$ is transformed by a scale factor into the assisted DLL code rate to maintain the DLL locking bandwidth when the carrier loop is locked. Therefore, the loop gain can be reduced and the anti-jamming capability of the code loop can be enhanced. However, the DLL with a closed-loop structure tracks drift error caused by the carrier Doppler-assistederror. When the carrier loop loses lock, the assisted mode is switched to the MEMS-SINS-assisted code loop. The MEMS-SINS velocity information corrected by IKF and the output of the code loop filter are combined into a driving signal to control the predicted signal delay. The code loop would only track the remaining frequency error. The code loop works in narrow band mode to enhance the loop’s anti-jamming capability.

Setting the PLL lock detector is used to detect the tracking state of the carrier phase. The output of the detector is a function of the carrier phase tracking error, expressed as

(28) $$L\_C2\phi_{k}=\frac{{\left(\sum_{i=1}^{m}I_{P,i}\right)}^{2}-{\left(\sum_{i=1}^{m}Q_{P,i}\right)}^{2}}{{\left(\sum_{i=1}^{m}I_{P,i}\right)}^{2}+{\left(\sum_{i=1}^{m}Q_{P,i}\right)}^{2}}$$

where $m=\frac{20\mathrm{ms}}{\mathrm{PIT}}$; PIT is the predetection integration time, and its value can be selected as 1, 2, 5, or 10 ms; $I_{P,i}$ is the prompt output of the in-phase branch; and $Q_{P,i}$ is the prompt output of the orthogonal branch. The threshold of the PLL lock detector is 0.7. The threshold is set when at least 85% of the signal energy is mainly in the in-phase I branch. If the output is less than 0.7, carrier loop lock loss occurs [20].

**Figure 7:**
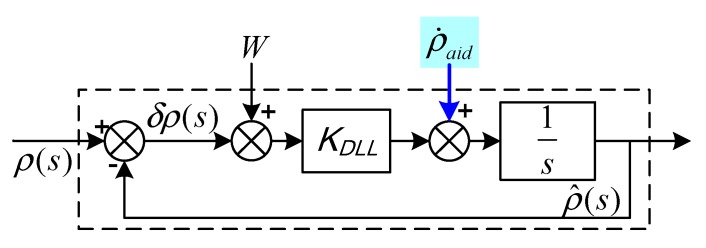
Model of PLL-assisted DLL tracking.

The PLL-assisted DLL mode includes a PLL frequency-assisted loop and a code tracking loop. Code loop-assisted information is calculated in the PLL frequency-assisted loop. The code tracking loop includes loop gain and noise bandwidth. The error model of PLL-assisted DLL tracking is shown in Figure 7.

The tracking error equation of the code loop can be expressed as:

(29) $$\delta {\dot{\rho }}_{DLL}=-K_{DLL}\delta \rho_{DLL}+\delta V_{aid}+K_{DLL}W$$

where δρ is the pseudo-range measurement error, $K_{DLL}$ is the code loop gain, W is the code loop driving noise caused by thermal noise and interference, and ${\dot{\rho }}_{aid}$ is the pseudo-range rate for assisted DLL.

Although the GPS signal is interrupted temporarily, as long as the carrier and code tracking are not separated and the assisted frequency is not interrupted, DLL could still provide valid synchronous code based on PLL frequency estimation assistance.

### 4.3. Modeling of the Innovative GPS/MEMS-SINS Ultra-Tight Integration

#### 4.3.1. State Equations

The error model of the innovative SINS/GPS ultra-tight integrated system includes the error models of SINS, GPS, PLL tracking, and DLL tracking.

##### SINS Error Model

SINS errors consist of position error, velocity error, misalignment angle error, and inertial measurement unit (IMU) error. The state equation is expressed as

(30) $${\dot{X}}_{I}=F_{I}X_{I}+G_{I}W_{I}$$

$$\begin{array}{l} X_{I}=[\begin{array}{ccccccccc} \delta L & \delta \lambda & \delta h & \delta V_{E} & \delta V_{N} & \delta V_{U} & \varphi_{E} & \varphi_{N} & \varphi_{U} \end{array}\\ {\begin{array}{ccccccccc} \varepsilon_{bx} & \varepsilon_{by} & \varepsilon_{bz} & \varepsilon_{rx} & \varepsilon_{ry} & \varepsilon_{rz} & \nabla_{x} & \nabla_{y} & \nabla_{z} \end{array}]}_{18\times 1}^{T} \end{array}$$

$$W_{I}={\left[\begin{array}{ccccccccc} \omega_{gx} & \omega_{gy} & \omega_{gz} & \omega_{bx} & \omega_{by} & \omega_{bz} & \omega_{ax} & \omega_{ay} & \omega_{az} \end{array}\right]}_{9\times 1}^{T}$$

where δL is latitude error; δλ is longitude error; δh is height error; $\delta V_{E}$, $\delta V_{N}$, and $\delta V_{U}$ are the east, north, and zenith velocity errors, respectively; $\varphi_{E}$, $\varphi_{N}$, and $\varphi_{U}$ are the misalignment angle errors in the east, north, and zenith directions, respectively; $\varepsilon_{b}$ is the standard deviation of the gyroscope measurement noise; $\varepsilon_{r}$ is the first-order Markov process of the gyroscope; ∇ is the constant bias of the accelerometer; $\omega_{g}$ is the angular random walk of the gyroscope; $\omega_{b}$ is the white noise in $\varepsilon_{r}$; $\omega_{a}$ is the random bias of the accelerometer; and $F_{I}$ and $G_{I}$ are determined by SINS error equations.

##### GPS Error Model

GPS errors consist of distance error caused by clock error $b_{clk}$ and clock frequency error $d_{clk}$. The state equation is expressed as

(31) $${\dot{X}}_{G}=F_{G}X_{G}+G_{G}W_{G}$$

$$X_{G}={\left[\begin{array}{cc} b_{clk} & d_{clk} \end{array}\right]}^{T},F_{G}=[\begin{array}{cc} 0 & 1\\ 0 & -\frac{1}{T_{clk}} \end{array}],G_{G}=I_{2}\;W_{G}={\left[\begin{array}{cc} \omega_{b} & \omega_{d} \end{array}\right]}^{T}$$

where $T_{clk}$ is the correlation time of the equivalent distance rate random walk, $\omega_{b}$ is the noise of the equivalent distance, and $\omega_{d}$ is the noise of the equivalent distance rate.

##### PLL Tracking Error Model

The PLL tracking error model can be expressed in matrix form as

(32) $$\left[\begin{array}{c} \delta \dot{\varphi }\\ \delta {\dot{f}}_{LPF} \end{array}\right]=\left[\begin{array}{cc} 0 & 2\pi \\ \frac{K_{d}}{\tau_{1}} & \frac{2\pi K_{d}\tau_{2}}{\tau_{1}} \end{array}\right]\left[\begin{array}{c} \delta \varphi \\ \delta f_{LPF} \end{array}\right]+\left[\begin{array}{c} 2\pi \\ \frac{2\pi K_{d}\tau_{2}}{\tau_{1}} \end{array}\right]\delta f_{dopp}$$

where $\delta \dot{\varphi }$ is the variation rate of carrier phase tracking error, $\delta {\dot{f}}_{LPF}$ is the variation rate of the loop filter output, $\delta f_{dopp}$ is the Doppler-assistederror, $K_{d}$ is the gain of the discriminator, and $\tau_{1}$ and $\tau_{2}$ are the time constants of the filter.

The error equations of the four tracking channels are merged to obtain the state equations of the PLL tracking error:

(33) $${\dot{X}}_{P}=F_{P}X_{P}+G_{P}W_{P}$$

where the state vector is chosen as $X_{P}={\left[\begin{array}{c} \delta \varphi_{1},\delta \varphi_{2},\delta \varphi_{3},\delta \varphi_{4},\delta f_{LPF1},\delta f_{LPF2},\delta f_{LPF3},\delta f_{LPF4} \end{array}\right]}^{T}$, $G_{P}$ is the system noise matrix, $W_{P}$ is the system noise vector, and $F_{P}$ is the system state matrix as follows:

$$F_{P}={\left[\begin{array}{cc} 0_{4\times 4} & 2\pi I_{4\times 4}\\ \frac{K_{d}}{\tau_{1}}I_{4\times 4} & \frac{2\pi K_{d}\tau_{2}}{\tau_{1}}I_{4\times 4} \end{array}\right]}_{8\times 8}$$

##### DLL Tracking Error Model

The state vector is chosen as $X_{D}=\left[\delta \rho_{DLL1},\delta \rho_{DLL2},\delta \rho_{DLL3},\delta \rho_{DLL4}\right]$, and the state equation of the DLL tracking error can be expressed as:

(34) $${\dot{X}}_{D}=F_{D}X_{D}+G_{D}W_{D}$$

where $W_{D}$ is the system noise vector, $G_{D}$ is the system noise matrix, and $F_{D}$ is the system state matrix:

$$F_{D}=-\;\left[\begin{array}{cccc} K_{DLL1} & & & \\ & K_{DLL2} & & \\ & & K_{DLL3} & \\ & & & K_{DLL4} \end{array}\right],\;G_{D}=\left[\begin{array}{cccc} K_{DLL1} & & & \\ & K_{DLL2} & & \\ & & K_{DLL3} & \\ & & & K_{DLL4} \end{array}\right].$$

Combining the error equations of the SINS, GPS, PLL tracking, and DLL tracking, the system state error equation can be expressed as

(35) $$\left[\begin{array}{c} {\dot{X}}_{I}\\ {\dot{X}}_{G}\\ {\dot{X}}_{P}\\ {\dot{X}}_{D} \end{array}\right]=\left[\begin{array}{cccc} F_{I} & & & \\ & F_{G} & & \\ F_{IP} & & F_{P} & \\ F_{ID} & & & F_{D} \end{array}\right]\left[\begin{array}{c} X_{I}\\ X_{G}\\ X_{P}\\ X_{D} \end{array}\right]+\left[\begin{array}{cccc} G_{I} & & & \\ & G_{G} & & \\ & & G_{P} & \\ & & & G_{D} \end{array}\right]\left[\begin{array}{c} W_{I}\\ W_{G}\\ W_{P}\\ W_{D} \end{array}\right]$$

where $F_{IP}$ and $F_{ID}$ are

$$F_{IP}=2\pi \frac{f_{L1}}{c}{\left[\begin{array}{c} {\vec{L}}_{i}\\ \frac{K_{d}\tau_{2}}{\tau_{1}}{\vec{L}}_{i} \end{array}\right]}_{8\times 18},\;F_{ID}={\left[\begin{array}{ccc} 0_{4\times 3} & {\vec{L}}_{i} & 0_{4\times 12} \end{array}\right]}_{4\times 18}^{}$$

where ${\vec{L}}_{i}$ is the direction cosine matrix (DCM) between satellites and the GPS receiver from the Earth-centered inertial (ECI) frame to the ECEF frame.

#### 4.3.2. Measurement Equations

Measurement equations consist of two parts: the difference between the GPS CSP $\rho_{s}$ and the pseudo-range $\rho_{I}$ of the observed satellite and the GPS receiver position calculated by MEMS-SINS, and the difference between TDCP velocity $V_{cp}$ and MEMS-SINS velocity $V_{I}$. The measurement vector, measurement noise vector, and measurement matrix of the integrated system are expressed as:

(36) $$\begin{array}{l} Z_{1}=\left[\rho_{I}^{j}-\rho_{s}^{j}\right]=H_{1}X+V_{1}\\ H_{1}=\left[\begin{array}{ccc} H_{\rho 1} & 0_{4\times 15} & \begin{array}{ccc} H_{\rho 2} & 0_{4\times 8} & {Ι}_{4} \end{array} \end{array}\right]\\ Z_{2}=\left[\begin{array}{c} V_{IE}-V_{cpE}\\ V_{IN}-V_{cpN}\\ V_{IU}-V_{cpU} \end{array}\right]=H_{2}X+V_{2}\\ H_{2}={\left[\begin{array}{ccc} 0_{3\times 3} & {Ι}_{3} & \begin{array}{ccc} 0_{3\times 12} & 0_{3\times 2} & 0_{3\times 12} \end{array} \end{array}\right]}^{} \end{array}\}$$

where *j* is the satellite number, $H_{\rho 1}$ is the conversion relation of the DCM between satellites and the GPS receiver from the ECI frame to the ECEF frame, $H_{\rho 2}$ is the relationship between GPS error and measurements, and $V_{1}$ and $V_{2}$ are the measurement noise matrices.

## 5. Experimental Verification and Analysis

To verify the performance of the innovative GPS/MEMS-SINS ultra-tight integration, highly dynamic and strong interference experiments were conducted on indoor semi-physical simulation experimental equipment. A land vehicle experiment was conducted outdoors.

The system uses low-precision MEMS-SINS. The IMU errors of MEMS-SINS are as follows: the constant bias and random bias of each accelerometer were chosen as $1\times 10^{-4}\;g$ and $5\times 10^{-5}\;g$(3σ), respectively; the standard deviation of each gyro measurement noise is 10°/h(3σ); and the angular walk random of each gyro is 5°/h(3σ). 

The carrier frequency of the L1 signal received by the GPS receiver antenna is 1575.42 MHz. After necessary amplification and local oscillator mixing, the carrier frequency is converted to an intermediate frequency. Finally, analog–digital converter (ADC) sampling with a frequency of 5.174 MHz is used to obtain the digital intermediate frequency signal with a theoretical intermediate frequency value of 1.405 MHz. The receiver uses quartz crystal as the timing frequency source, and its daily frequency stability is 10^−11^. The noise bandwidth of the PLL carrier loops is 4 Hz, the second-order code loop bandwidth is 1 Hz, the correlator spacing is 10 sampling intervals, and PIT is 1 ms. The updating frequencies of the two subfilters are 1 Hz and 100 Hz. The updating frequency of the senior filter is 1 Hz.

### 5.1. Semi-Physical Simulation Experiment 

**Figure 8:**
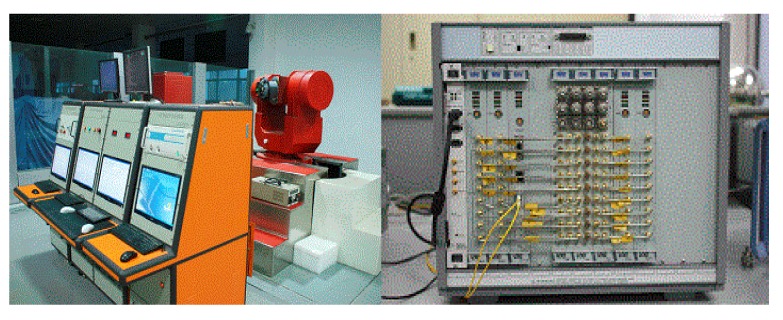
Simulation experiment system setup.

Figure 8 depicts the semi-physical simulation experiment system, which was composed of a master control computer, a numerical simulation computer, a three-axis simulation platform, a satellite simulator, a jammer, and measurement and control equipment. The system can be used to simulate and reproduce the air movement environment in the laboratory and to test, verify, and evaluate the performance of the integrated navigation system.

The numerical simulation computer generates the flight path of the aircraft and the standard flight path of the missile according to the selected aircraft motion model, missile dynamics model, and motion model, as well as the relative motion model between the aircraft and the missile, seeker model, sensor model, steering gear model, control signal generation model, and other models. The numerical simulation computer takes the standard flight path of the missile as the evaluation benchmark of the whole inertial navigation performance test. The gyroscope and accelerometer outputs are obtained, and the triaxial turntable is controlled by the master control computer to generate corresponding measurement outputs of the gyroscope. The acceleration information is input to the integrated navigation products. The missile’s standard flight path information is fed into the satellite simulator and jammer through the numerical simulation computer to generate digital satellite signals and interference signals corresponding to the input flight path. In the operation of the onboard integrated navigation system, and the output subtracts the standard trajectory to evaluate the accuracy of integrated navigation.

### 5.2. Result and Analysis of the High-Precision GPS Measurement Model

#### 5.2.1. Result and Analysis of CSP

**Figure 9:**
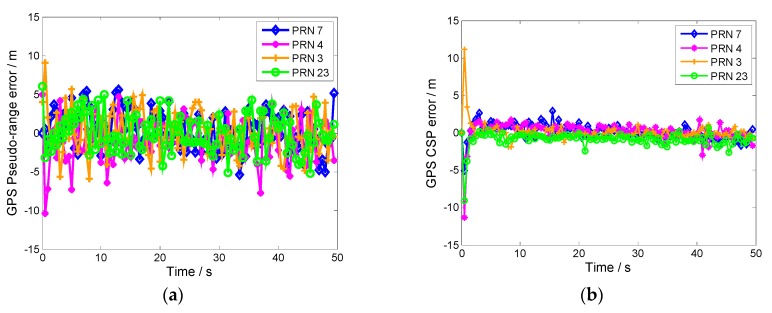
(**a**) GPS pseudo-range measurement error. (**b**) GPS carrier-smoothed pseudo-range (CSP) measurement error.

High-precision navigation measurements can be obtained by CSP. The errors of the GPS conventional pseudo-range and CSP are depicted in Figure 9a,b, respectively.

The CSP measurements are more accurate than those of the conventional pseudo-range because code measurement does not compensate for the errors caused by the satellite clock, ephemeris, ionosphere, and troposphere. However, the propagation error can be compensated for by code measurements combined with carrier phase measurements. The Hatch filter assigns greater weighting to carrier phase measurements than code measurements. The multipath error of the code measurement could be smoothed within the filter length range. The CSP measurements provide more accurate IKF measurements.

#### 5.2.2. Result and Analysis of TDCP Velocity

**Figure 10:**
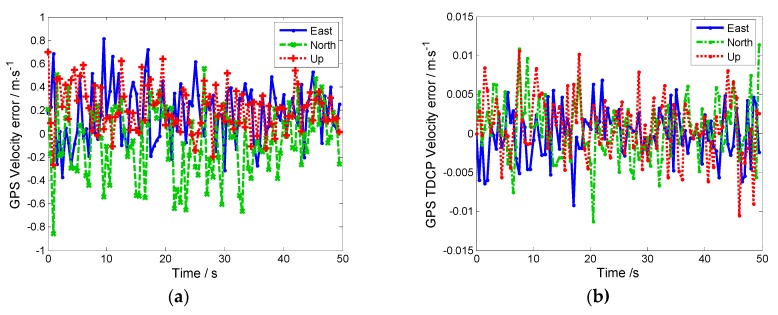
(**a**) GPS velocity measurement error. (**b**) GPS TDCP velocity measurement error.

The high-precision velocity of the aircraft can be calculated using high-precision carrier phase measurements. The GPS TDCP velocity and GPS conventional velocity are shown in Figure 10a,b, respectively.

As seen in Figure 10, due to the GPS tracking loop being aided by the Doppler-assisted information, the dynamic tracking ranges of the carrier loops and the carrier loop noise bandwidth are reduced. The tracking accuracy of the GPS tracking loop is improved. Therefore, the accuracy of TDCP velocity can reach the centimeter level and even the millimeter level. Compared to GPS conventional velocity, the updating frequency of TDCP is higher to help the GPS tracking loops. 

### 5.3. Result and Analysis of the Tracking Loop in Innovative Ultra-Tight Integration

#### 5.3.1. Performance Analysis Under Highly Dynamic Conditions

**Figure 11:**
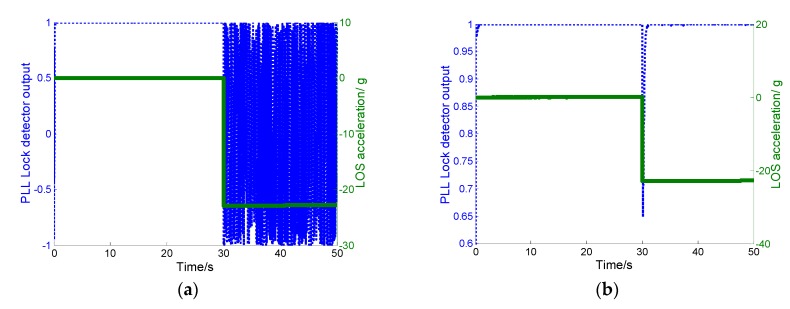
(**a**) PLL lock detector output of conventional ultra-tight integration. (**b**) PLL lock detector output of our proposed ultra-tight integration.

In the simulation, the aircraft experienced a step acceleration of 30 *g* from 30 s to 50 s, which corresponds to a step acceleration of −22.8 *g* along the LOS vector for channel 2. The simulations of conventional and ultra-tight integration under these conditions are shown in Figure 11a,b, respectively.

As seen in Figure 11, the PLL lock detector output is represented on the left-hand-side y-axes. The LOS acceleration is represented on the right—hand-side y-axes; the PLLs of both the conventional and ultra-tight integrations are in lock-in state from 0 to 30 s (when the PLL lock detector outputs are greater than 0.7). However, the PLL of the conventional ultra-tight integration immediately lost lock at the initial moment of the step acceleration, mainly because the major navigation error in MEMS-SINS leads to a deviation in the GPS-assisted information when the aircraft undergoes highly dynamic maneuvers. The tracking error of PLL increases, thereby increasing the GPS measurement error. Then, the estimate precision of the IKF decreases and the accuracy of correcting MEMS-SINS error also decreases. The deviations in the Doppler-assistedinformation are not effectively corrected promptly, resulting in PLL lock loss and steady state error of the carrier phase occurring beyond the tracking threshold. In Figure 11a, PLL lock detector output oscillates within [−1,1] after 30 s, indicating that continuous cycle slips occur during the step acceleration. In contrast, there is a transient tracking error in TDCP velocity-assisted PLL at 30 s, but PLL cycle slips never occur. The tracking error is rapidly reduced. Then, tracking step acceleration of 22.8 *g* can be achieved.

#### 5.3.2. Performance Analysis Under Severe Jamming Conditions

In the simulation, wideband noise was injected into GPS intermediate frequency (IF) signals from 20 to 40 s. The $C/N_{0}$ of each channel decreased linearly from the conventional 42 dB·Hz to 5 dB·Hz. The wideband noise disappeared at 40 s. The $C/N_{0}$ returned to the conventional level.

**Figure 12:**
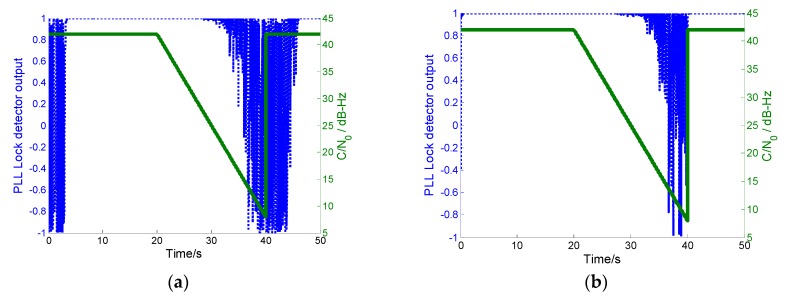
(**a**) PLL lock detector output of the conventional ultra-tight integration. (**b**) PLL lock detector output of our proposed ultra-tight integration.

The PLL lock detector outputs of the conventional and innovative ultra-tight integrations are shown in Figure 12a,b, respectively. With increasing the energy of the jamming signal, the PLL lock detector output of the conventional integration decreases gradually. Once the detector output is lower than 0.7, PLL loses lock. When the jamming signals disappear, PLL lock delay results in importing error. In contrast, once the PLL of the proposed integration loses lock under increasing jamming energy, the system operation mode is automatically switched to MEMS-SINS-assisted DLL. In this mode, the noncoherent integration of the received signal and the local carrier wave generated by the MEMS-SINS-derived frequency turns the carrier wave of the received signal off. The code-phase error information is provided for the DLL discriminator. Thus, DLL can output measurements. With the relatively superior anti-jamming capability of DLL, the MEMS-SINS-assisted DLL mode can maintain navigation performance, even when $C/N_{0}$ is lower than 8 dB·Hz in the short term. The anti-jamming capability of the innovative integration is enhanced, and this makes the integration more robust and available. After the jamming signal disappeared, the PLL lock detector output returned to the lock-in state rapidly. After short-term interference, the MEMS-SINS-assisted DLL mode is sufficient to provide carrier frequency information within the tracking pull-in range for PLL. Therefore, in this mode, PLL re-locks the received signal rapidly and accurately, eliminating the need to implement coarse acquisition for the carrier.

### 5.4. Land Vehicle Experiment

**Figure 13:**
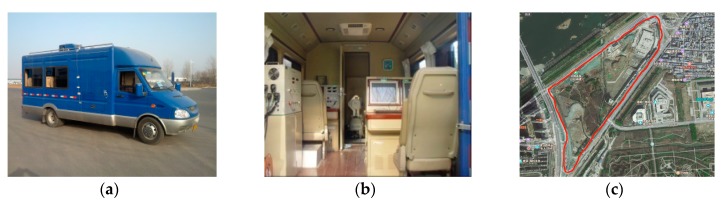
Test platform. (**a**) Mobile test vehicle, (**b**) internal test equipment, and (**c**) trajectory on map shown by the red line.

**Figure 14:**
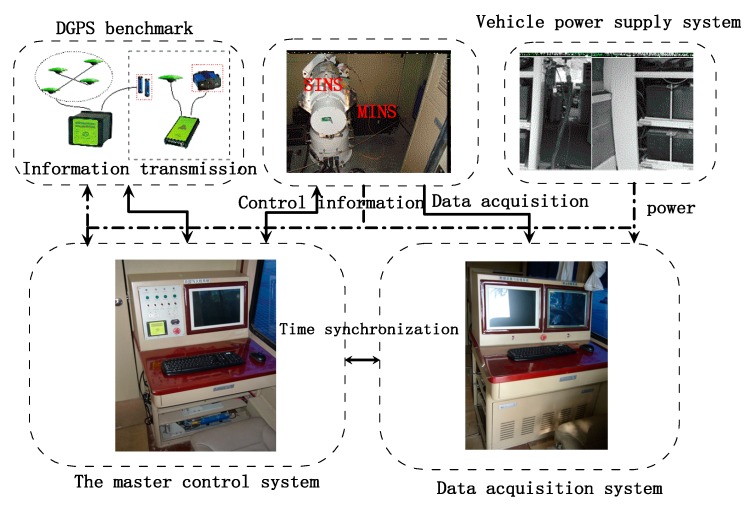
Test platform block diagram.

The land vehicle system is shown in Figure 13 and Figure 14. As the test benchmark, the land vehicle had a master inertial navigation system (MINS) and a high-precision differential global positioning system (DGPS), which employed JNSGyro-4T produced by JAVAD, the positioning accuracy of which can reach 1 cm. The land vehicle system was controlled by the master control system, and the integrated navigation system was installed on a vehicle-mounted turntable. The integrated navigation system was initialized by the master inertial navigation system, and the data acquisition system recorded the output of the integrated navigation system and test benchmark. The difference between MINS-DGPS and SINS after the lever arm and time delay compensation could be used to evaluate the navigation accuracy. Figure 13c shows the dynamic trajectory of the vehicle obtained from the GPS.

### 5.5. Result and Analysis of Our Proposed Ultra-Tight Integration

**Figure 15:**
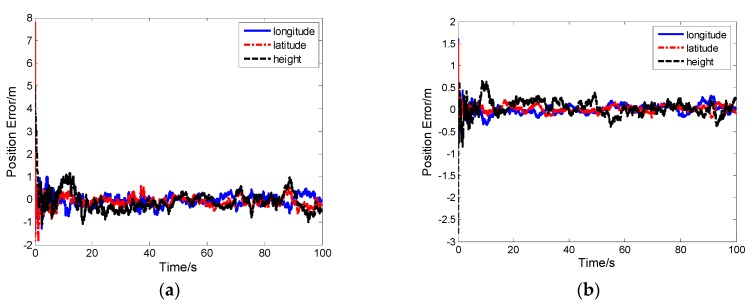
(**a**) Position error of the conventional ultra-tight integration. (**b**) Position error of our proposed ultra-tight integration.

**Figure 16:**
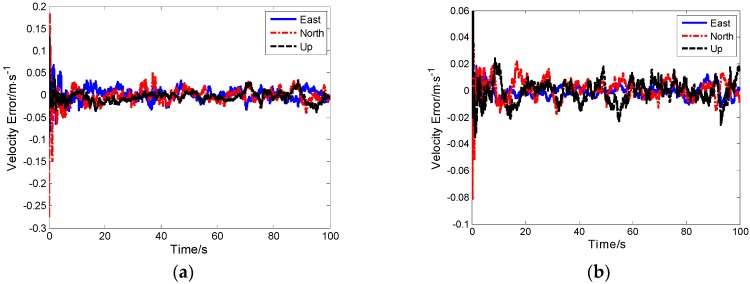
(**a**) Velocity error of the conventional ultra-tight integration. (**b**) Velocity error of the innovative ultra-tight integration.

As seen in Figure 15 and Figure 16, the proposed ultra-tight integration is more accurate under the same simulation conditions because the tracking of the proposed integration system aided by high-precision TDCP velocity is more accurate compared with conventional MEMS-SINS ultra-tight integration. Improving tracking accuracy ensures that the carrier phase information output is more accurate. Thus, a cycle is formed, improving the GPS tracking performance, assisted information, and the measurement of IKF. The position error and velocity error of the proposed integration system converge rapidly, and navigation accuracy is further improved.

## 6. Conclusions

The navigation performance of ultra-tight GPS/MEMS-SINS integration is influenced by the accuracy of MEMS-SINS. To improve the navigation performance of GPS/MEMS-SINS ultra-tight integration, we proposed an innovative GPS/MEMS-SINS ultra-tight integration scheme based on using high-precision carrier phase measurement as the IKF measurement. According to the scheme design and simulation experiments, we drew the following conclusions:

(1) The dynamic tracking range of carrier loops is reduced by assisting PLL with TDCP velocity. The loop equivalent bandwidth is enhanced so that the carrier loop noise bandwidth can be greatly reduced to restrain the thermal noise. The dynamic performance, anti-jamming capability, and the tracking accuracy of PLL are improved.
(2) The Doppler range is reduced by assisting DLL with PLL and MEMS-SINS. The loop gain and the code correlator spacing are reduced. The anti-jamming capability and the tracking accuracy of DLL are improved.
(3) With the improved tracking accuracy, carrier phase measurements maintain high precision. MEMS-SINS errors are accurately estimated and corrected using the TDCP velocity and CSP as the IKF measurements. The navigation accuracy of the innovation GPS/MEMS-SINS ultra-tight integration was thus further improved.

Our proposed ultra-tight integration system has the potential for broad use in engineering applications that use low-cost MEMS-SINS for high-precision navigation. The dynamic performance and anti-jamming capability of our proposed system are improved compared with conventional ultra-tight integration.
